# The association between serum total folic acid concentration and severe difficulty falling asleep in US adults: NHANES 2005–2008

**DOI:** 10.3389/fneur.2023.1225403

**Published:** 2023-09-21

**Authors:** Haoyu An, Qiyun Xue, Jingyi Zhang

**Affiliations:** ^1^School of Life Science and Technology, China Pharmaceutical University, Nanjing, Jiangsu, China; ^2^Faculty of Science, The University of Hong Kong, Hong Kong SAR, China; ^3^School of Science, China Pharmaceutical University, Nanjing, Jiangsu, China

**Keywords:** folic acid, neurotransmitter, insomnia, difficulty falling asleep, National Health and Nutrition Examination Survey

## Abstract

**Objective:**

The present study aimed to explore the correlation of serum total folic acid concentration with severe difficulty falling asleep among US adults.

**Methods:**

Cross-sectional data were collected from the National Health and Nutrition Examination Survey (NHANES) from 2005 to 2006 and 2007 to 2008 cycles. Participants were divided into the severe difficulty falling asleep (SDFA) group and the control group according to the monthly frequency of having difficulty falling asleep. Serum total folic acid was taken as independent and dependent variables, respectively. The correlation of serum total folic acid concentration with severe difficulty falling asleep was examined using multivariable logistic regression models, where the adjusted odds ratio (OR) and 95% confidential intervals (CIs) were calculated.

**Results:**

Overall, 8,926 individuals from the NHANES 2005 to 2006 and 2007 to 2008 waves were included in the analysis, of whom 683 participants had severe difficulty falling asleep. Higher serum total folic acid concentration (ng/ml) was associated with lower odds of severe difficulty falling asleep after adjusting for potentially confounding factors (OR = 0.98; 95% CI: 0.97–1.00), while participants at the highest quartile had the least odds of severe difficulty falling asleep (OR = 0.55; 95% CI: 0.40–0.74). The subgroup analysis based on gender, smoking history, and diabetes showed that this negative correlation was more significant in males, smokers, and nondiabetic population after adjusting for confounding factors.

**Conclusion:**

High levels of serum folic acid were significantly related to less odds of severe difficulty in falling asleep among US adults, suggesting that folic acid supplementation may be beneficial to the prevention and even treatment of severe difficulty falling asleep.

## 1. Introduction

According to The International Classification of Sleep Disorders, 3rd ed., (ICSD-3), insomnia is defined as difficulty falling asleep, difficulty staying asleep, and early awakening, which happens at least three times a week for a month (1). It was estimated to affect up to 50% of the adult population globally (2). In addition to increasing the risk of mental health issues, drug and alcohol addiction, healthcare utilisation, insomnia can lead to daytime exhaustion, distress, and cardiovascular diseases. As a result, it is of vital importance to study those with insomnia, especially difficulty falling asleep which has not received much attention before.

Folic acid, also called vitamin B9, is a crucial nutrient that plays essential roles in various physiological functions, such as neurotransmitter production, DNA synthesis, and cell division (3). Several studies have investigated the link between serum total folic acid concentration and insomnia, but the findings are inconsistent. While one cross-sectional study found an independent inverse association between sleep disturbance and serum folic acid (OR = 0.72; 95% CI: 0.58–0.91, *p* = 0.008) (4), another cross-sectional study that focused on individuals aged 65 years or older did not find statistical significance regarding the association of insomnia severity status and serum folic acid levels (*p* > 0.05) (5).

To simplify the research question, the present study concentrated solely on difficulty falling asleep and defined severe difficulty falling asleep as over 15 times of difficulty falling asleep situation per month. The data from the National Health and Nutrition Examination Survey (NHANES) were analyzed to explore the risk factors of severe difficulty falling asleep and examine the clinical relevance between serum total folic acid concentration and severe difficulty in falling asleep among US adults. We suspected that some factors such as gender, unhealthy living habits may be covariates, so we did the correction and stratified analysis to control the impact.

## 2. Materials and methods

### 2.1. Data source

NHANES is a program aimed at assessing the health and dietary condition of both adults and children across the United States. The Ethics Review Board of the National Center for Health Statistics (NCHS) authorized the informed written consent provided by all participants. Our study analyzed the data collected from NHANES between 2005 and 2008 in a cross-sectional approach.

### 2.2. Study population

NHANES monitors the health and nutritional status of adults and children across the United States and provides cross-sectional data. We used variable – ridageyr (age at screening adjudicated – recode) to screen American adults over 20 years from 2005 to 2006 and 2007 to 2008 demographic data. Participants all received serological examinations and answered questionnaires on demographics, life behaviors, disease information, and sleep conditions.

Inclusion criteria:individuals from the NHANES between 2005 and 2008, over 20 years of age. Exclusion criteria: (1) missing data on the outcome—frequency of difficulty falling asleep; (2) missing data on serum folic acid; and (3) missing data on covariates.

**Figure 1:**
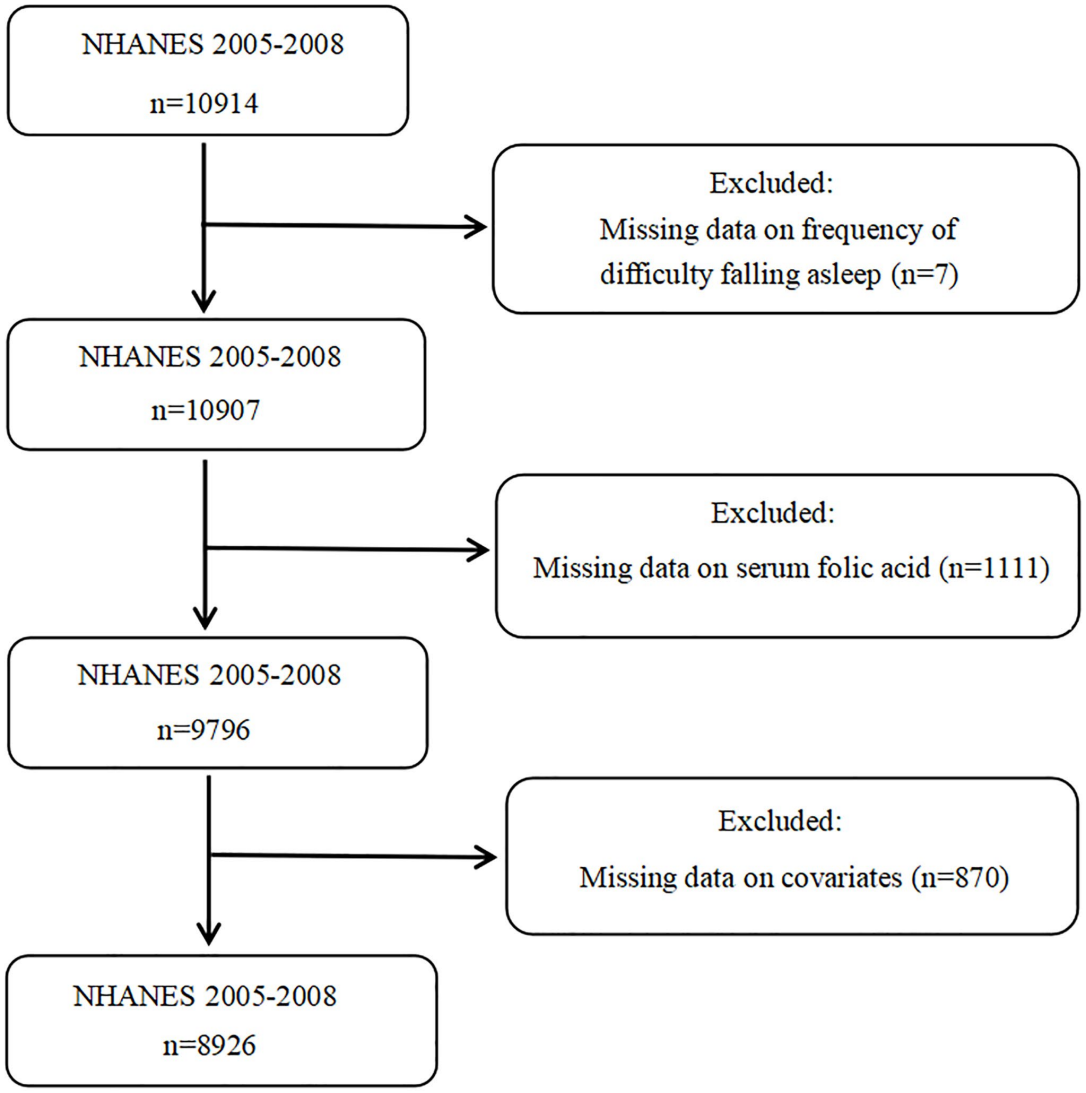
Flowchart of the participants.

A total of 20,497 participants completed the interview in selected survey cycles and 10,914 of them aged 20 years or older. The presence or absence of difficulty in falling asleep was determined as the outcome of the investigation, and seven participants missing this data were excluded. A total of 1,111 of the remaining 10,907 participants missed the data on serum folic acid levels and were excluded. The remaining 9,796 participants were then reviewed to exclude 870 participants whose data on covariates we wanted to consider were not available. Finally, 8,926 participants were enrolled in the final analysis (Figure 1).

### 2.3. Questionnaire data assessment

A subject was considered to have severe difficulty falling asleep if he or she answered almost always (≥15 times per month) to the question “How often do you have difficulty falling asleep?” Serological samples came from specimen donors, then they were processed, stored under −20°C conditions, and shipped to Centers for Disease Control and Prevention, Atlanta, GA for analysis. Compared with previous bio-rad laboratories “Quantaphase II folic acid” radioassay kit, in 2007–2008, NHANES used microbiological assays to to estimate the concentration of folate in serum. First of all, diluted serum was added to an assay medium containing *Lactobacillus rhamnosus* and all of the nutrients necessary for the growth of *L. rhamnosus* except folate. Then the inoculated medium was incubated for 45 h at 37°C, the growth of *L. rhamnosus* was proportional to the amount of total folate present in serum or whole blood samples. The folate level could be assessed by measuring the turbidity of the inoculated medium at 590 nm in a PowerWave X340 Microplate reader. The following sociodemographic information was collected through a structured questionnaire: age, education level (less than high school, high school graduate/GED or equivalent, some college or AA degree, college graduate or above), race and ethnic groups (non-Hispanic white, non-Hispanic Black, other race or ethnic groups). Other race or ethnic groups included other Hispanic, Mexican American and other race-including multi-racial. Besides, the questionnaire also includeed marital status (married/living with partner, widowed/divorced/separated, never married) and BMI calculated as weight (kg) divided by height squared (kg/m^2^). The body measurement data were collected by trained health technicians according to the Anthropometric Standardization Reference Manual (6).

Data on alcohol use status was collected from the question “Had at least 12 alcohol drinks per year?”(yes/no). Similarly, the information about smoking history was collected based on the answers to the question “Have you smoked at least 100 cigarettes in your entire life?” (yes/no), where those who responded with a “yes” were rated as having a positive smoking history.

Besides, our study gathered some information about diseases such as hypertension and diabetes because they were considered as potential factors affecting sleep quality. Questionnaire questions were as follows: “Have you ever been told by a doctor or health professional that you have diabetes or sugar diabetes?” and “Have you ever been told by a doctor or other health professional that you had hypertension, also called high blood pressure?”

### 2.4. Statistical analysis

The study described the distribution of serum total folic acid concentration in SDFA group and non falling asleep problem group (control group). Then, it analyzed the differences between the two groups using weighted chi-square tests for categorical variables and weighted logistic regression models for continuous variables. “Wtmec4yr” was selected as the weight variable, calculated by the formula: “wtmec4yr” = 1/2 × “wtmec2yr.” The link between serum total folic acid and severe difficulty in falling asleep was examined, for which multivariable logistic regression models were utilized to calculate the odds ratios (ORs) and 95% confidential intervals (CIs). Three multivariable logistic regression models were constructed, with serum folic acid levels grouped into quartiles and the lowest quartile used as the reference category. Subgroup analyses were also performed based on gender, smoking history, and diabetes.

The present study expressed continuous variables as means ± standard deviation (SD) and categorical variables as frequency (percentage). The statistical analyses were conducted using package R (www.R-project.org; version 3.4.3). A *p*-value ≤ 0.05 was considered statistically significant.

## 3. Results

**Figure 2:**
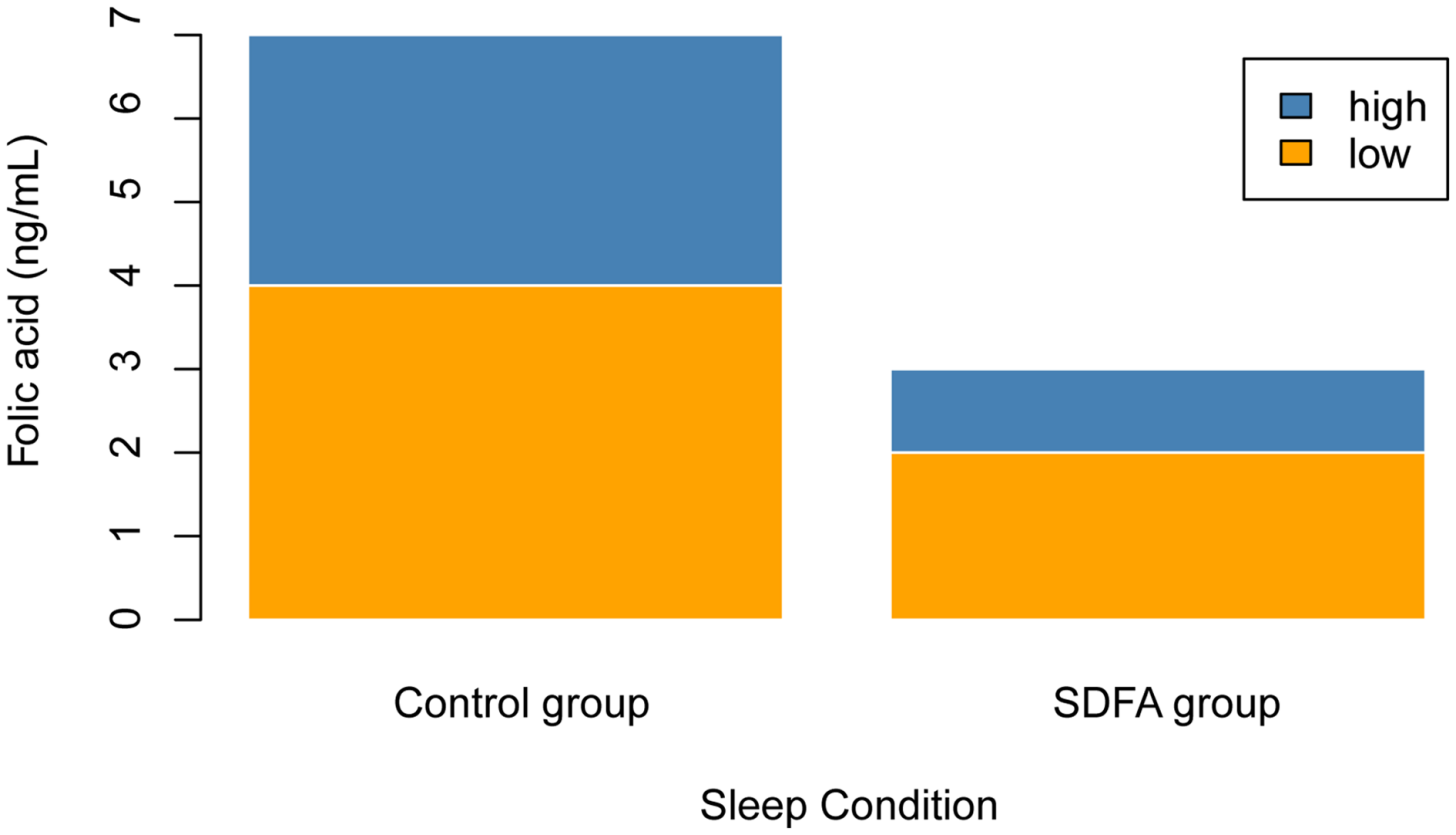
Histogram of the serum folate concentration.

Figure 2 illustrates that a greater proportion of participants in the SDFA group had low serum folate concentrations compared with the control group. Table 1 presents the baseline characteristics of participants. Of the 8,926 participants (52% females and 48% males; mean (SD) age, 46.8 [16.68] years), 7.65% had severe difficulty falling asleep. In the two groups with or without severe difficulty falling asleep, variables including gender, education level, marriage status, smoking history, hypertension, diabetes, and serum folic acid were all significantly different. Participants with severe difficulties in falling asleep had lower serum folic acid levels and were more likely to be female, live alone, and have positive smoking history, high blood pressure, or diabetes.

**Table 1:** Baseline characteristics of participants, NHANES 2005–2008.

		Severe difficulty falling asleep		
Characteristics	Overall	No (*n* = 8,243)	Yes (*n* = 683)	*p* value
Age(years)	46.80 ± 16.68	46.84 ± 16.73	46.34 ± 16.10	0.8798
Gender, *n* (%)^a^				<0.001
Male	4,343 (48)	4,084 (49)	259 (35)	
Female	4,583 (52)	4,159 (51)	424 (65)	
Race/ethnicity, *n* (%)^a^				0.5
Non-hispanic white	4,448 (72)	4,092 (72)	356 (73)	
Non-hispanic black	1814 (10)	1,677 (10)	137 (11)	
Mexican American/other hispanic/other race	2,664 (17)	2,474 (17)	190 (16)	
Education level, *n* (%)^a^				<0.001
Below high school	2,552 (18)	2,288 (18)	264 (28)	
High school grad/GED or equivalent	2,164 (25)	1989 (24)	175 (30)	
College or AA degree	2,432 (31)	2,256 (31)	176 (31)	
College graduate or above	1778 (26)	1710 (28)	68 (11)	
Marriage status, *n* (%)^a^				0.017
Never married	1,380 (16)	1,279 (16)	101 (15)	
Married/living with partner	5,542 (66)	5,147 (66)	395 (62)	
Widowed/divorced/separated	2004 (19)	1817 (18)	187 (24)	
Alcohol, *n* (%)^a^				0.4
No	2,686 (25)	2,481 (25)	205 (27)	
Yes	6,240 (75)	5,762 (75)	478 (73)	
Smoking history, *n* (%)^a^				<0.001
No	4,650 (52)	4,393 (53)	257 (35)	
Yes	4,276 (48)	3,850 (47)	426 (65)	
BMI group, *n* (%)^a^				0.071
<25	2,615 (32)	2,438 (33)	177 (30)	
25–30	3,063 (34)	2,859 (34)	204 (31)	
≥30	3,150 (34)	2,861 (33)	289 (40)	
Hypertension, *n* (%)^a^				<0.001
No	5,908 (70)	5,529 (70)	379 (60)	
Yes	3,018 (30)	2,714 (30)	304 (40)	
Diabetes, *n* (%)^a^				0.035
No	7,886 (92)	7,310 (92)	576 (88)	
Yes	1,040 (8.2)	933 (8.0)	107 (12)	
Serum folic acid (ng/mL)	16.82 ± 11.63	16.92 ± 11.59	15.50 ± 12.00	0.0352

Values are means ± SD for continuous variables. *p* value was calculated by weighted logistic regression model for continuous variables and weighted chi-square test was performed for catagorical variables.

a Unweighted frequency counts and weighted percentages are shown.

Three multivariable logistic regression models were constructed in the present study (Table 2). The global *p* value for model 1, model 2, and model 3 were 0.048, 0.022 and 0.043. Model 1 did not have any covariates adjusted, while Model 2 had age, gender, and race/ethnicity as adjusted covariates, and Model 3 was adjusted for age, gender, race/ethnicity, education level, marital status, alcohol use, smoking status, BMI, hypertension, and diabetes. Among the three models, Model 2 revealed a link between severe difficulty in falling asleep and serum folic acid concentration among the participants included in the study (OR = 0.98; 95% CI: 0.97–1.00). Compared with those at the lowest quartile of serum folic acid level, participants at the highest quartile were found to have reduced odds of severe difficulty in falling asleep (OR = 0.55, 95% CI, 0.40–0.74; *p* trend <0.001). These results suggest that individuals with greater levels of serum folic acid were less likely to have severe difficulty in falling asleep.

**Table 2:** Association between serum folic acid and severe difficulty falling asleep, NHANES 2005–2008.

	Model 1	Model 2	Model 3
	OR (95%)	OR (95%)	OR (95%)
Serum folic acid (ng/ml)	0.99 (0.98, 1.00)	0.98 (0.97, 1.00)	0.99 (0.98, 1.00)
Serum folic acid (ng/ml, quartile)			
Q1 (0.70–9.50)	Referrence	Referrence	Referrence
Q2 (9.50–13.90)	0.69 (0.52, 0.91)	0.67 (0.51, 0.90)	0.77 (0.56, 1.05)
Q3 (13.90–20.20)	0.65 (0.50, 0.85)	0.60 (0.46, 0.80)	0.72 (0.54, 0.97)
Q4 (20.20–209.30)	0.62 (0.47, 0.81)	0.55 (0.40, 0.74)	0.70 (0.51, 0.96)
*p* for trend	<0.001	<0.001	0.021

Model 1: no covariates were adjusted. Model 2: age, gender, and race/ethnicity were adjusted. Model 3: age, gender, race/ethnicity, education level, marital status, alcohol use, smoking status, BMI, hypertension, and diabetes were adjusted.

The subgroup analysis based on gender (Table 3), in both Model 1 and Model 2, appeared to illustrate a more significant inverse association between serum folic acid concentration and severe difficulty in falling asleep among males. However, when participants were stratified by smoking history and diabetes, this association was not significant among non-smokers and participants with diabetes.

**Table 3:** Association between serum folic acid and severe difficulty falling asleep by gender, smoking history, and diabetes.

	Model 1	Model 2	Model 3
	OR (95%)	OR (95%)	OR (95%)
Stratified by gender^a^			
Male	0.97 (0.95, 0.99)	0.97 (0.95, 0.99)	0.98 (0.96, 1.00)
Female	0.99 (0.97, 1.00)	0.99 (0.97, 1.00)	0.99 (0.98, 1.01)
Stratified by smoking history			
No	1.00 (0.99, 1.01)	0.99 (0.97, 1.01)	1.00 (0.98, 1.01)
Yes	0.98 (0.97, 1.00)	0.98 (0.97, 1.00)	0.99 (0.97, 1.00)
Stratified by diabetes			
No	0.98 (0.97, 1.00)	0.98 (0.97,0.99)	0.99 (0.98, 1.00)
Yes	1.00 (0.98, 1.02)	1.00 (0.97, 1.02)	1.00 (0.97, 1.02)

Model 1: no covariates were adjusted. Model 2: age, gender, and race/ethnicity were adjusted. Model 3: age, gender, race/ethnicity, education level, marital status, alcohol use, smoking status, BMI, hypertension, and diabetes were adjusted.

a In the subgroup analysis by gender, the model was not adjusted for the stratification variable itself.

## 4. Discussion

The prevalence of insomnia varies among different populations. Ohayon (2) estimated that approximately 30 to 50% of the global adult population experiences symptoms of insomnia. The pathological features of insomnia include changes in the brain’s neurotransmitters (7), hyperarousal of the central nervous system (8), and alterations in circadian rhythm (9). Certain medications, such as benzodiazepines and non-benzodiazepine hypnotics, can be prescribed to treat insomnia (10), but only under the guidance of healthcare providers due to potential side effects.

Nowadays, studies have suggested that maintaining adequate serum folic acid levels may promote sleep health. For example, Grandner et al. found that patients with low serum folic acid levels had more severe sleep disturbances, impaired sleep continuity, and decreased sleep efficiency (11). Similarly, in our analysis, an inverse association was found between serum total folic acid concentration and severe difficulty in falling asleep among American adults. Moreover, the subgroup analysis based on gender revealed that this association was more significant in males, while the subgroup analyses based on smoking history and diabetes showed that this association was significant only among participants who had a positive smoking history or did not have diabetes.

The biological mechanism behind the association of serum folic acid levels with difficulty in falling asleep is complex and still controversial. Current research suggests that folic acid’s role in neurotransmitter synthesis and circadian rhythm regulation might be two main mechanisms. In the first hypothesis, folic acid is involved in synthesizing neurotransmitters, such as serotonin and melatonin, which are known to regulate sleep–wake cycles (12). Specifically, serum folic acid takes part in the synthesis of 5-hydroxytryptophan (5-HTP), a precursor of serotonin (13). Serotonin, in turn, is a precursor to melatonin (14), the hormone responsible for regulating sleep. Therefore, low serum folic acid levels might lead to an imbalance in these neurotransmitters and contribute to difficulty falling asleep. In the other hypothesis, folic acid has been reported to influence circadian rhythms (15). Circadian disruption may lead to sleep disturbances such as delayed sleep phase syndrome, affecting the ability to fall asleep at a desired time. It is plausible that altered serum folic acid levels might modulate circadian functioning, thereby impacting sleep quality. However, more comprehensive research is needed to further explore additional possibilities and investigate the dose–response relationship between serum total folic acid levels and difficulty in falling asleep.

Our study has several strengths. Firstly, the large and representative sample size enhances the credibility and stability of the findings. Secondly, we have taken into account multiple factors that could potentially impact the association between difficulty falling asleep and serum total folic acid levels.

However, the limitations of this study should be noted. First, despite adjusting for several covariates, there may be other unmeasured variables that may bias the results. Second, our study is a cross-sectional design, preventing us from establishing a causal relationship between serum total folic acid levels and severe difficulty in falling asleep. Therefore, more large-scale cohort studies are needed in the future to verify the impact of serum total folic acid levels on difficulty in falling asleep or insomnia. Third, the different laboratory methods of 2005–2006 and 2007–2008 may had potential impact on the data, and how did this impact the results are still unclear. The last but not the least, residual confounding effects of some unstratified factors and multiple comparisons issues may remained in the observed treatment-outcome effect.

## 5. Conclusion

To sum up, our study indicates that lower serum total folic acid levels were found to be linked with a higher risk of severe difficulty in falling asleep among American adults, especially in women, those who live alone, and those with a positive smoking history, hypertension, or diabetes. These results could offer new perspectives on how to manage insomnia, but more research is necessary to validate them.

## Data availability statement

Publicly available datasets were analyzed in this study. This data can be found here: Centers for Disease Control and Prevention (CDC), National Center for Health Statistics (NCHS), National Health and Nutrition Examination Survey (NHANES), https://wwwn.cdc.gov/nchs/nhanes/Default.aspx, NHANES 2005–2006 and NHANES 2007–2008.

## Ethics statement

Ethical review and approval was not required for the study on human participants in accordance with the local legislation and institutional requirements. Written informed consent from the patients/participants or patients/participants’ legal guardian/next of kin was not required to participate in this study in accordance with the national legislation and the institutional requirements.

## Author contributions

HA: conceptualization, methodology, and supervision. HA and QX: formal analysis and investigation. HA, QX, and JZ: writing original draft preparation and commented on previous versions of the manuscript. QX: writing review and editing. JZ: resources. All authors contributed to the article and approved the submitted version.
